# Portosystemic shunt surgery for severe portal hypertension due to portal thrombosis after bariatric surgery

**DOI:** 10.1093/jscr/rjae254

**Published:** 2024-04-24

**Authors:** Natalia Reyes, Alessandra Jarufe, Eduardo Briceño, Eduardo Viñuela, Jorge Martínez, Martin Dib, Nicolás Jarufe

**Affiliations:** Department of Hepatobiliary Surgery, P. Universidad Católica de Chile, Hospital Clínico UC CHRISTUS, Santiago 8330024, Chile; Department of Hepatobiliary Surgery, P. Universidad Católica de Chile, Hospital Clínico UC CHRISTUS, Santiago 8330024, Chile; Department of Hepatobiliary Surgery, P. Universidad Católica de Chile, Hospital Clínico UC CHRISTUS, Santiago 8330024, Chile; Department of Hepatobiliary Surgery, P. Universidad Católica de Chile, Hospital Clínico UC CHRISTUS, Santiago 8330024, Chile; Department of Hepatobiliary Surgery, P. Universidad Católica de Chile, Hospital Clínico UC CHRISTUS, Santiago 8330024, Chile; Department of Hepatobiliary Surgery, P. Universidad Católica de Chile, Hospital Clínico UC CHRISTUS, Santiago 8330024, Chile; Department of Hepatobiliary Surgery, P. Universidad Católica de Chile, Hospital Clínico UC CHRISTUS, Santiago 8330024, Chile

**Keywords:** portosystemic shunt, bariatric surgery, portal hypertension

## Abstract

Portal vein thrombosis is a rare complication after laparoscopic sleeve gastrectomy, a widely performed bariatric surgery procedure. Occasionally, the development of portal vein thrombosis can progress to more severe conditions, including portal hypertension and cavernomatosis, thereby presenting a complex and challenging clinical scenario. The management of such complications often requires careful consideration; however, surgical intervention in the form of a splenorenal shunt is an exceptional indication. We present the case of a 33-year-old female patient who had previously undergone laparoscopic sleeve gastrectomy in 2014 and subsequently developed portal thrombosis, followed by cavernomatosis and associated complications of portal hypertension. A proximal splenorenal shunt procedure and splenectomy were successfully performed to manage portal hypertension. The presentation of this clinical case aims to contribute to the available evidence and knowledge surrounding this rare and challenging pathology.

## Introduction

Portal vein thrombosis (PT) is a relatively uncommon but potentially severe complication that can occur after laparoscopic surgeries. It is seen in about 5–15% of cases of mesenteric ischemic events [1]. In the context of laparoscopic bariatric surgery, PT is considered rare, with an incidence of 0.5% [2]. This complication may be attributed to a hypercoagulable and inflammatory state in these patients.

PT can progress over time, leading to portal cavernomatosis, which is characterized by multiple venous collaterals that replace the portal vein in the hepatic pedicle. Cavernomatosis can result in symptoms and complications related to portal hypertension. Managing portal hypertension can be challenging, and with the advancements in liver transplantation and the emergence of endovascular and endoscopic treatment methods, surgical interventions are now reserved for selected cases. Among the surgical options, the splenorenal shunt is often preferred for certain patients [3].

We present a case of a patient who developed portal cavernomatosis following laparoscopic sleeve gastrectomy. A proximal splenorenal shunt was performed. Our goal is to contribute to the limited evidence regarding this rare postoperative complication, as no published cases in the medical literature have undergone surgical treatment after PT with cavernomatosis in sleeve gastrectomy.

## Case report

The case of a 33-year-old female patient with a history of obesity, BMI of 40.1 is presented. She underwent laparoscopic sleeve gastrectomy in 2014 in another center, resulting in a weight loss of 15 kg. However, just 2 weeks after the surgery, due to abdominal pain, a CT scan confirmed PT. The etiological investigation identified secondary thrombophilia related to protein C and S deficiencies. As a consequence, she was placed on anticoagulant and beta-blocker therapy due to the presence of esophageal varices on upper gastrointestinal endoscopy that was made for the study of possible complications.

**Figure 1:**
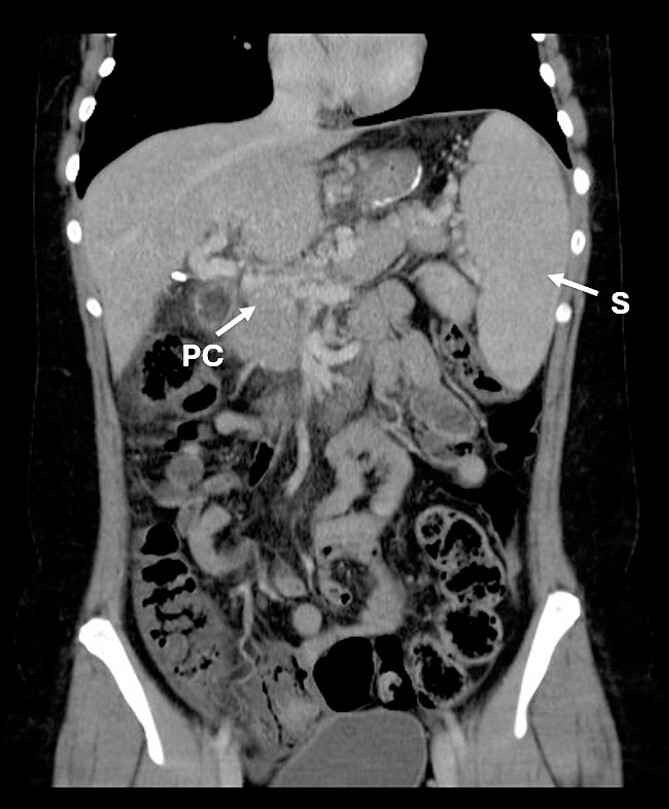
CT scan showing portal cavernomatosis and massive splenomegaly. PC, portal cavernomatosis; S, splenomegaly.

Three years after the bariatric surgery, during a routine imaging follow-up, the presence of portal cavernomatosis was detected (Fig. 1), along with significant palpable giant symptomatic splenomegaly, which subsequently led to thrombocytopenia and recurrent episodes of gingival bleeding. Furthermore, gastroesophageal varices had enlarged significantly, posing a high risk of bleeding and requiring recurrent endoscopic banding.

The findings of abdominal portosystemic collaterals, observed by upper gastrointestinal endoscopy and imaging studies, confirmed the presence of portal hypertension.

**Figure 2:**
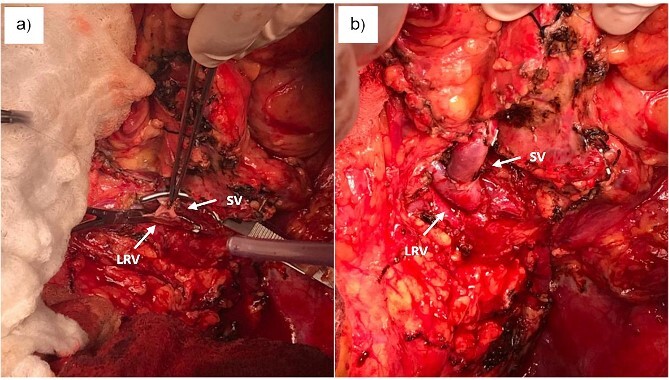
(a) Creation of proximal splenorenal shunt between clamps. (b) Proximal splenorenal shunt. SV, splenic vein; LRV, left renal vein.

Given the complexity of these multiple secondary complications, the patient was referred to our center and underwent a multidisciplinary evaluation. Following a comprehensive analysis, a proximal splenorenal shunt procedure for the management of portal hypertension was proposed. The procedure included splenectomy and end-to-side anastomosis between the splenic vein and left renal vein (Fig. 2). Intraoperative Doppler monitoring was conducted, and post-procedure angiography confirmed good shunt patency. Patient was discharge on the seventh post-operative day without complications and instructions to continue anticoagulant therapy.

Four years after the splenorenal shunt procedure, the patient successfully achieved a full-term pregnancy. An upper digestive endoscopy revealed no evidence of esophageal varices, and no signs of portal hypertension were observed during the medical follow-up.

## Discussion

Diagnosing PT in the postoperative period can be challenging due to its limited symptomatology; thus, the incidence is underestimated [2].

Several risk factors are involved in the development of PT after bariatric surgery. Pneumoperitoneum has been proposed as a cause of increased intra-abdominal pressure and consequent ectasia of blood flow, apart from the endothelial damage that could occur in the manipulation of vessels of the portal system during sleeve gastrectomy. Additionally, there are medical factors associated with hypercoagulability, such as the presence of hidden neoplasms, the use of oral contraceptives, and systemic thrombophilic conditions like protein C and S, as well as factor XII deficiency [4–6].

Giannis et al. found that 34.9% of gastric sleeve patients had thrombophilias as a common condition. However, no statistically significant association with PT was confirmed (p 0.61). Conversely, advanced age and low center volume showed significant variables, p-values of 0.02 and < 0.0001, respectively [2]. In the present case, several of these factors contributed to the development of portal thrombosis.

Anticoagulation is the primary treatment for PT post-bariatric surgery [2] and is safe and effective in patients with esophageal varices [7]. Surgical indication depends on the thrombosis extent and intestinal ischemia. Limited evidence supports thrombectomy, thrombolysis in occlusive thromboses and intestinal resection in mesenteric ischemia [8–10].

In this case, anticoagulation managed PT without short-term complications. However, long-term consequences included cavernous transformation of the portal vein, portal hypertension and associated complications.

The management of portal hypertension complications from thrombosis and cavernomatosis rarely requires surgery. Medical measures, endoscopic and occasionally interventional radiology, are usually sufficient. However, in cases like this, with massive splenomegaly causing thrombocytopenia and symptoms like abdominal discomfort and frequent gingival bleeding, surgical intervention becomes the most viable long-term solution [3].

Choosing the type of surgical shunt to perform is also a challenge. One of the most frequently used is the Warren or distal splenorenal shunt, which has the advantage of being selective and, therefore, lowers the risk of post-operative encephalopathy [11]. In this case, a proximal shunt with splenectomy was preferred to enhance platelet count and alleviate the discomfort and abdominal pain due to her giant splenomegaly. Since chronic liver damage was absent, the risk of encephalopathy with this nonselective shunt was minimal. The patient’s long-term prognosis has been favorable, with a high quality of life, including an uncomplicated pregnancy and delivery, without the need for further procedures. Therefore, surgical intervention for portal hypertension due to cavernomatosis in patients without chronic liver damage is effective, requiring specialized centers and multidisciplinary discussions.

It is important to note that no additional published evidence resembles this case. Hence, we hope to contribute to medical knowledge to care for these challenging cases most effectively.

In conclusion, portal thrombosis after bariatric surgery is a well-described complication that can have serious long-term consequences in cases where portal permeabilization with anticoagulants is not achieved. In the case of failure of other medical and interventional therapies, surgery can improve these consequences. Therefore, surgery continues to have a role in selected cases of portal hypertension in the setting of a liver without cirrhosis.
